# Professional identity formation (PIF) – What has NKLM 2.0 to say about it? A workshop report

**DOI:** 10.3205/zma001828

**Published:** 2026-03-23

**Authors:** Jonathan Ahles, Sandra Apondo, Pascal O. Berberat, Barbara Jömann, Rolf Kienle, Claudia Kiessling, Daniela Mauer, Götz Fabry

**Affiliations:** 1 University of Freiburg, Faculty of Medicine, Dean’s Office, Freiburg, Germany; 2 Technical University of Munich (TUM), TUM School of Medicine and Health, Department Clinical Medicine, TUM Medical Education Center, TUM University Hospital, Munich, Germany; 3 Ruhr University Bochum, Institute of General Practice and Family Medicine (AM RUB), Bochum, Germany; 4 Charité – Universitätsmedizin Berlin, Vice Dean’s Office for Studies and Teaching, Berlin, Germany; 5 Witten/Herdecke University, Faculty of Health, Chair for the Education of Personal and Interpersonal Competencies in Health Care, Witten, Germany; 6 University of Bonn, Faculty of Medicine, Dean’s Office, Bonn, Germany; 7 University of Freiburg, Faculty of Medicine, Institute for Medical Psychology and Medical Sociology, Freiburg, Germany

**Keywords:** professional identity formation, professional identity development, professional conduct, leadership and management, NKLM, understanding of roles

## Abstract

**Objective:**

Medical education should support the development of a professional identity. However, it is still unclear what exactly is meant by professional identity formation (PIF), how it proceeds, and how it can be specifically supported. Against this background, this workshop report describes an attempt by a group of experts to identify and evaluate learning objectives in NKLM 2.0 that could be relevant to PIF. The aims of this project were, on the one hand, to determine the extent to which PIF has been incorporated into the NKLM and, on the other hand, to analyze how PIF can be understood on the basis of PIF related learning objectives.

**Methodology:**

In a multi-stage, iterative process, a subgroup (N=6) of the GMA Committee on Professional Identity Formation identified PIF-relevant learning objectives in the NKLM and derived fundamental topics and distinctions from them.

**Results:**

Basically, a categorization into essential and associated learning objectives emerged, although the assessments of the participants regarding the assignment to the categories varied greatly. 130 learning objectives were marked and weighted as essential, 14 of which were classified as particularly relevant. The result was a weighted list of essential and associated learning objectives for the topic of PIF, as well as two fundamental, summarizing learning objectives in the areas of “role understanding” and “self-reflection”.

**Discussion:**

The selection and evaluation of learning objectives within the group were often heterogeneous. This illustrates that the topic of PIF has been vaguely defined to date and is obviously understood differently. In order to explicitly map PIF in the NKLM, the underlying learning objectives would have to be formulated more precisely. In addition, a theory-based definition of PIF would be helpful in order to revise the essential learning objectives and specify the boundaries of the topic.

## Zusammenfassung

**Zielsetzung:**

Das Medizinstudium soll auch die Entwicklung einer professionellen Identität unterstützen. Allerdings ist bislang unklar, was genau unter einer professionellen Identitätsentwicklung (Professional Identity Formation, PIF) verstanden wird, wie sie sich vollzieht und wie sie gegebenenfalls gezielt unterstützt werden kann. Vor diesem Hintergrund beschreibt dieser Werkstattbericht den Versuch, durch eine Gruppe von Expert:innen Lernziele im NKLM 2.0 zu identifizieren und zu bewerten, die für die PIF relevant sein könnten. Ziele waren zum einen festzustellen, in wieweit PIF Eingang in den NKLM gefunden hat und zum anderen zu analysieren, wie PIF sich auf Grundlage dieser Lernziele verstehen lässt.

**Methodik:**

In einem mehrstufigen, iterativen Prozess identifizierte eine Untergruppe (N=6) des GMA Ausschusses „Professional Identity Formation“ PIF-relevante Lernziele des NKLM und leitete grundlegende Themenbereiche und Unterscheidungen davon ab.

**Ergebnisse:**

Grundsätzlich ergab sich eine Kategorisierung in essenzielle und assoziierte Lernziele, wobei die Einschätzungen der beteiligten Akteur:innen über die Zuordnung zu den Kategorien sehr unterschiedlich ausfiel. 130 Lernziele wurden als essenziell markiert und gewichtet, 14 davon als besonders relevant eingestuft. Als Ergebnis entstand eine gewichtete Liste essenzieller und assoziierter Lernziele für das Thema PIF sowie zwei grundlegende, zusammenfassende Lernziele zu den Bereichen „Rollenverständnis“ und „Selbstreflexion“.

**Diskussion:**

Auswahl und Bewertung der Lernziele innerhalb der Gruppe waren häufig heterogen. Dies zeigt, dass das Themenfeld PIF bisher unscharf definiert ist und offensichtlich unterschiedlich verstanden wird. Um PIF im NKLM explizit abzubilden, müssten zugrundeliegende Lernziele genauer formuliert werden. Zusätzlich ist eine theoriebasierte Begriffsbestimmung von PIF hilfreich, um die essenziellen Lernziele zu überarbeiten und die Grenzen des Themenfeldes zu konkretisieren.

## 1. Introduction

Medical training should not only focus on acquiring specialist knowledge and technical skills, but should also help future doctors to develop a professional attitude and identity [1], [2]. Of particular importance in this regard is conscious engagement with the specific characteristics of the medical profession, e.g., the norms, values, and role expectations described in the professional code of conduct (in Germany: (Muster-)Berufsordnung), self-critical reflection on professional and personal socialization, and awareness of one's own subjective state and intersubjective relationships with others, especially patients, their relatives, and other professional groups [3]. In the international discussion, the term "professional identity formation" (PIF) has been used for some time to describe how these aspects can be integrated into medical education, training, and continuing education, and which teaching and learning formats are best suited for this purpose [1], [4], [5]. Currently, the international debate on PIF is strongly influenced by Anglo-American authors. Although there is no uniform definition of PIF, it is often described as an active and constructive process of identity formation [6], [7], [8], which is characterized by the examination and (critical) adoption of characteristics, traits, values, and norms of the medical profession and leads to a person “feeling, thinking, and acting like a physician” [1], [2]. For a long time, scientific debate in German-speaking countries was dominated by social science studies that took an analytical and critical look at the process of medical socialization and the concept of medical identity [9], [10]. However, with the development of the National Competence-Based Catalog for Learning Objectives in Medicine (NKLM) [11], which explicitly envisages the role of the physician as a professionally acting person, more pragmatic and prescriptive questions have arisen, especially how and what medical studies can and should contribute to the development of a professional medical identity. Against this background, the Professional Identity Formation (PIF) Committee of the DACHJ Association for Medical Education (GMA) was founded in 2021 [12]. This article is the workshop report of a selection and evaluation process of learning objectives from the NKLM that are potentially relevant to PIF, which was initiated at a closed meeting of the committee in 2022. 

A key challenge in addressing the topic of PIF is that there is no uniform definition of what exactly is meant by professional identity or identity development [13], [14]. If the term is defined very broadly, then in principle the entirety of medical training can be understood as professional identity development, since technical expertise is also a central aspect of professionalism. As a rule, however, the term is defined much more narrowly, focusing on aspects that are primarily related to adopting the role of a physician or medical socialization [15]. 

Due to this definitional uncertainty, a subgroup of the PIF committee is working on a discursive definition of PIF for German-speaking countries [15].

Despite the ongoing discussion about a more precise definition of PIF, learning objectives were nevertheless defined during the development of the NKLM [https://nklm.de/zend/menu] that explicitly refer to some of the frequently described characteristics of professional identity development (NKLM 2.0: IV.2.7 “The physician as a professional” or chapter VIII.6 “Professional Conduct, Ethics, History, and Medical Law”). For example, the NKLM graduate profile under IV.2.7 defines physicians as “acting professionally by expressing empathy and professional distance, altruism and self-care, as well as critical reflection and efficiency”. An example from chapter VIII.6 is learning objective VIII.6-01.2.9, which requires graduates to “recognize ethical conflicts, analyze them, and deal with them professionally in practice”. In addition, the catalog also contains learning objectives in other sections and chapters that – depending on the underlying understanding – may be important for professional identity formation (e.g., in chapters VIII.2 “medical communication” or VIII.3 “interprofessional cmpetencies”). In chapter VIII.2, for example, learning objective VIII.2-02.1.2. defines that graduates “adopt a patient-centered (congruent, accepting, and empathetic) attitude, communicate accordingly, and [are able to] professionally manage closeness and distance”.

Thus, given the definitional problems described above, when developing NKLM 2.0, the learning objectives were not systematically derived on the basis of a pre-existing construct definition for “professionalism” or “professional identity (formation)”, but were pragmatically negotiated in a multi-stage consensus process based on the learning objectives of NKLM 1.0. Like similar frameworks for competence-based training, NKLM 1.0 was also developed through such a consensus process involving a large number of experts [16], [17]. What the experts involved in this process understand by “professional”, for example, what theoretical foundations they refer to, and whether they also had a specific concept of professional identity formation in mind remains open. Although it can be assumed that PIF is considered an important training goal for medical education, it has not been directly included in the NKLM as such. Thus, there are neither learning objectives that explicitly refer to the concept of PIF, nor does the catalog directly refer to the medical education discourse surrounding PIF. Furthermore, the catalog does not indicate whether and, if so, in what way, reference was made to any conceptual or empirical work on the subject of PIF when the learning objectives were drawn up.

Against this background, the GMA PIF Committee came up with the idea of searching the NKLM for learning objectives that could be relevant on the basis of the broad preliminary understanding of PIF outlined above as a process of actively engaging with the physician’s role or medical socialization, which leads to feeling, thinking, and acting like a physician. This was intended to clarify the following questions: To what extent and in what way is the topic of PIF (explicitly) addressed in the NKLM? Which NKLM learning objectives are relevant to PIF from the perspective of those involved? Can the analysis and discussion of the NKLM learning objectives provide a clearer picture of what professional identity development entails?

Overall, the process described here is also an attempt to make the concept of PIF, which has so far only been implicitly included in the NKLM learning objectives, more concrete by examining these objectives. This approach can thus be understood as complementary to the attempt to first define PIF more precisely, e.g., on the basis of conceptual and normative considerations, and then to derive learning objectives from this [18], [19]. A more concrete and thus more visible definition of PIF is particularly important for the revision of the NKLM so that important core elements of the concept are retained in future revisions.

This article presents the various work steps and results in the form of a "workshop report" and discusses their significance for the integration of PIF into medical education, training, and continuing education. 

## 2. Method

**Figure 1:**
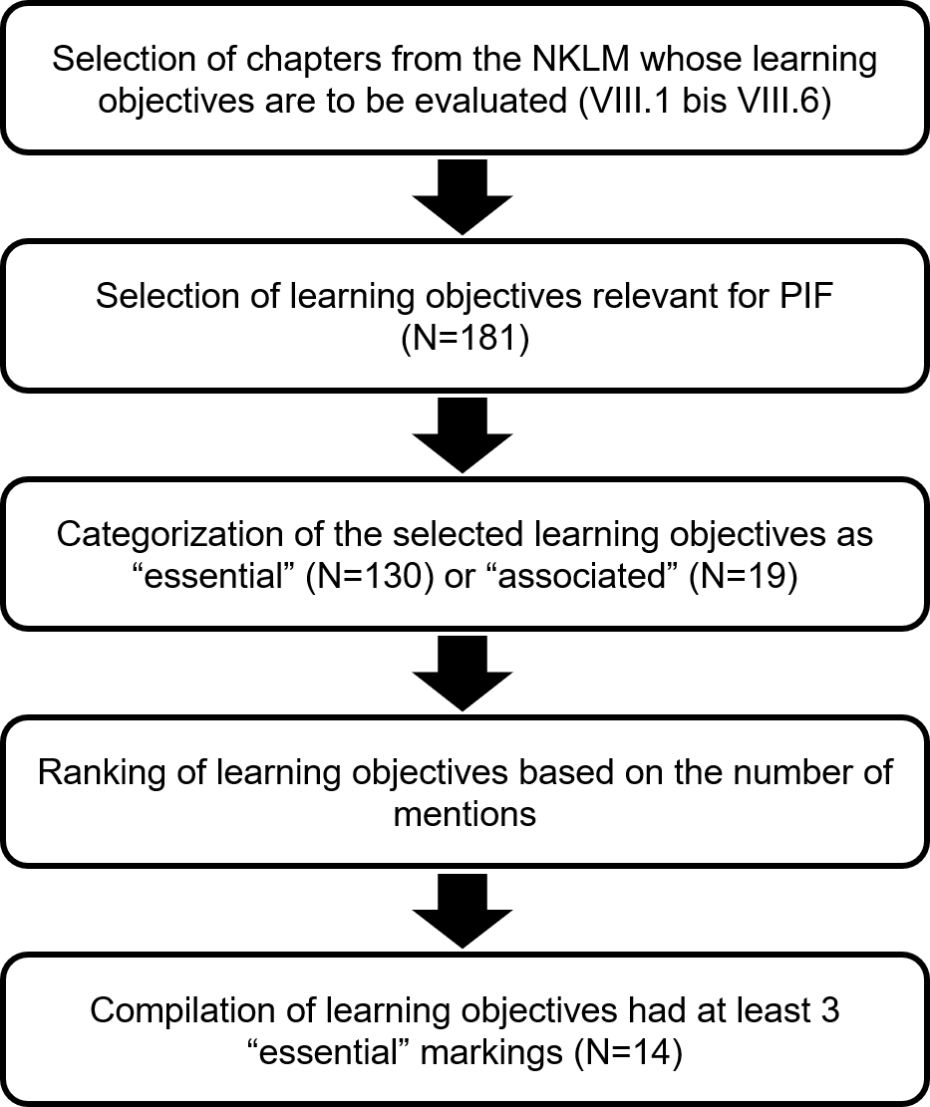
Procedure for selecting and evaluating the NKLM learning objectives (version 2.0) for “Professional Identity Formation”

The procedure was divided into three steps, which are described individually below (see figure 1 (Fig. 1)). The aim was to use a Delphilike approach to gradually arrive at consensual, expert-based results [20]. 

### 2.1. Selection of NKLM chapters to be evaluated

Based on an initial discussion in a working group meeting of the GMA Committee on Professional Identity Formation (PIF), the chapters of NKLM 2.0 [https://nklm.de/zend/menu] were identified in which PIF-relevant learning objectives were most likely to be found. The chapters in which the higher-order competencies for medical education are defined (VIII.1-VIII.7) were selected, although the chapters on clinical skills (VIII.7) were not taken into account because they tend to define rather instrumental-technical learning objectives. A sub-working group (UAG) consisting of members of the committee was formed to continue the work. All members of the UAG have specialist expertise, as they are directly responsible for the design, planning, and implementation of teaching in medical education in various functions and at various medical schools, and have also dealt intensively with the topic of PIF. Due to the exploratory approach, no further definitional restrictions were made apart from the preliminary understanding of PIF described in the introduction. The aim was therefore not to examine whether or to what extent the learning objectives found in the NKLM correspond to a specific definition or model of PIF. Rather, the exploratory approach was intended to provide information about which aspects of PIF are explicitly or implicitly addressed in the NKLM, which learning objectives of the NKLM are considered relevant to PIF by the participants, and whether this process can provide a more precise picture of what PIF entails.

### 2.2. Collection of PIF-relevant learning objectives

The learning objectives of the selected NKLM chapters (N=532) were made available to all members of the working group in an workable format (Excel file). First, each of the individual experts (N=6) selected from these 532 learning objectives those that they considered to be fundamentally relevant to PIF. The individually selected learning objectives were compiled in another Excel spreadsheet. This resulted in a collection of learning objectives (N=181) that the experts considered relevant to PIF in general.

### 2.3. Categorization and weighting of the selected learning objectives 

An initial analysis of the learning objectives selected in the first step showed that there were obviously different ideas within the UAG about which aspects are relevant to PIF; some learning objectives had been selected by several experts, others only by single persons (see figure 1 (Fig. 1)). In addition, it became apparent during the discussion that not all of the selected learning objectives were considered equally important for PIF by all participants. In order to obtain a clearer picture, the working group members were therefore asked to rank order all the learning objectives selected in the first step according to their relevance for the PIF. The learning objectives could be rated as either “essential” (mark: e) or “associated” (mark: a). The label “essential” was to be assigned to learning objectives that were considered particularly important or indispensable for the PIF. The label “associated” was to be assigned if the learning objective was related to PIF but not necessarily considered central to it.

The evaluations from this second round, in which six experts participated again, were summarized and compiled in a file (see attachment 1 ). All of the learning objectives mentioned are listed there with the number of mentions (see 2.2), the number of “essential” markings, and the number of “associated” markings. Learning objectives that were neither rated as “essential” nor “associated” were not processed any further.

In an additional step, the learning objectives were weighted based on the “essential” markings. The learning objectives that had received at least three “essential” markings were selected as particularly relevant to the topic of PIF and as a basis for further discussion.

## 3. Results

Chapters VIII.1-VIII.6 of the NKLM [16] contain a total of 532 learning objectives. Of these, 181 learning objectives were selected in the first step as fundamentally relevant to PIF (see table 1 (Tab. 1)). In the second step, 130 of the 181 learning objectives were marked as essential. The number of markings ranged from one to five (see table 2 (Tab. 2)). Most of the learning objectives marked as essential come from NKLM chapters VIII.3 (Interprofessional Competencies), VIII.5 (Leadership and Management), and VIII.6 (Professional Conduct and Ethics, History and Medical Law). Thirty-eight learning objectives were marked as associated. It is noteworthy here that almost all of these learning objectives (37/38) were marked as essential by only one person, and only one was marked as essential by two people. It is also striking that half of the learning objectives marked as associated (19/38) were marked as essential by other experts (see table 2 (Tab. 2) and attachment 1 ).

A total of 14 learning objectives were marked as essential by three to five people; with one exception, they are taken from chapters VIII.5 (Leadership and Management) and VIII.6 (Professional Conduct and Ethics, History and Medical Law). One learning objective marked as essential three times was found in chapter VIII.3 (Interprofessional Competencies) (see attachment 2 ).

The assessments for learning objectives from chapters VIII.2 (Medical Communication) and VIII.3 (Interprofessional Competencies) were less clear-cut. In the discussions among the committee members, it became clear that although both areas are considered central to successful medical practice, they are not perceived as specific or central aspects of PIF, but rather as topics associated or related to PIF. Similar remarks were also made for other learning objectives from the subject areas of ethics and history of medicine, time- and self-management, and health economics.

## 4. Discussion

As a key result of the process described here, it can first be noted that various aspects formulated as learning objectives in NKLM 2.0. [https://nklm.de/zend/menu] are considered by experts to be relevant for the professional identity development of medical students. A significant proportion of the learning objectives found in the chapters on the higher-order competencies for medical education are considered relevant to the PIF by the experts involved here. Two categories can be distinguished (see table 2 (Tab. 2)):

1. *Essential learning objectives: *These learning objectives are considered particularly important for PIF and clearly define important core competencies for PIF. The learning objectives on which consensus was most easily reached here deal primarily with (self-)reflection on the role of the physician and interaction with patients (see attachment 2 ). Most of them can be found in those chapters of the NKLM 2.0 (VIII.5 Leadership and Management and VIII.6 Professional Conduct and Ethics, History and Medical Law) that already suggest a priori proximity to PIF-relevant aspects; however, individual learning objectives from other chapters were also mentioned.
2. *Associated learning objectives:* This category of learning objectives is less well defined, especially since there is even less consensus in the evaluation than in the first category. It contains many learning objectives that are also considered essential by individual experts. The uncertainty about which aspects (can) contribute to PIF is even more evident here.

The results show that there are hardly any learning objectives that are considered equally important by all or even a majority of experts. In particular, the assessment of individual learning objectives as essential or associated varied greatly from person to person. Overall, the majority of learning objectives (145) were considered relevant by only one or two (out of six) experts. The designation as “essential” also showed a wide spread, with most learning objectives (116) being named by only a minority of experts (one to two out of six). Nineteen learning objectives were rated as both essential and associated, i.e., some were considered fundamental to PIF and some were considered only peripherally relevant. The differences in assessment are remarkable in that the people involved in this process are all members of the GMA PIF committee and already have prior knowledge and experience of teaching and research in the field of PIF. The heterogeneity of the assessments did not diminish during the process of discussing the learning objectives, but became even more apparent with the attempt to divide the learning objectives into essential and associated ones. In this respect, it can be assumed that this heterogeneity is inherent in the current state of discussion in German-speaking countries and its complexity, and cannot be attributed to a lack of content knowledge [8]. A first interim conclusion could therefore be that various aspects of PIF are currently reflected in various learning objectives of the NKLM. In the absence of a concrete and agreed definition of PIF, teaching experts assign a heterogeneous weighting to the relevance of these learning objectives.

The discussion also revealed that, in the opinion of the UAG, the learning objectives available in the NKLM do not yet cover all important aspects of PIF. Against this background, an attempt was therefore made to formulate two fundamental learning objectives based on the weighted list of learning objectives, which could serve as a consensus of the aspects of PIF discussed in the UAG. They combine as many of the learning objectives most frequently rated as essential as possible and can thus be understood as a synthesis or reformulation of these learning objectives:

1. Understanding of roles [Synthesis of VIII.3-02.1.1, VIII.6-03.1.1, and VIII.6-03.1.4]: [VIII.5-11.1: They practice and develop substantial skills of self-reflection and self-awareness. They are able to...]
2.
  1. practice structured and outcome-oriented reflection on their own medical role (including tasks, functions, and responsibilities) in encounters with patients and colleagues from other health professions.
  2. identify their own values, norms, and personality traits that are relevant to their professional identity formation and apply these appropriately in different kinds of relationship.
3. Self-reflection [Synthesis of VIII.6-03.1.1 and VIII.6-03.1.4]: [VIII.6-03.1: They are capable of self-reflection. They are able to...]
4.
  1. observe their attitudes, competencies, and actions, critically analyze them themselves and in exchange with others, and, if necessary, change their behavior in the sense of an outcome-oriented reflection process.

Based on the existing learning objectives of the NKLM, the result of the UAG's work is, on the one hand, a weighted list of essential learning objectives for the topic of PIF and, on the other hand, two very fundamental and comprehensive learning objectives that summarize the discussion of the topic. 

## 5. Outlook

The apparent differences in understanding PIF and in assessing possible relevant learning objectives also prompted a fundamental discussion of the understanding of professionalism and PIF. The blurred boundaries of the topic of PIF led the experts to engage in an intensive discourse on these “border areas” in particular. This was accompanied by the question of the extent to which PIF should be understood less as a narrowly defined construct of explicitly teachable and learnable competencies and more as a certain attitude. This attitude would then either manifest itself in the realization of certain typical competencies, as defined in NKLM chapters VIII.5 and VIII.6, for example, or even permeate the exercise of all medical competencies as a whole. In the latter case, the question would be how PIF can be represented at all within the framework of a competency-based catalog of learning objectives [21]. Defining PIF in terms of an attitude rather than an operationalizable competency does not necessarily have to be a disadvantage, as defining it as an attitude could lead to greater emphasis on the topic, since PIF would then have to be taken into account in all competency domains of the NKLM [16]. In addition, “attitudes” are often understood and used as an essential domain of learning objectives alongside “knowledge” and “skills” [22]. However, attitudes and learning objectives in the affective domain (according to Bloom's taxonomy) have so far been less well operationalized and less explicitly taught and tested than the other two domains [23], [24], [25]. The PIF discourse could offer a new opportunity here and contribute to the necessary specification of the attitudinal dimensions or affectively accentuated learning objectives (Bloom) in medical education [23].

This leaves open the question of the extent to which the heterogeneous assessment within the UAG is due to the content and structure of the NKLM or is inherent in the topic of PIF itself. On the one hand, the aspects of PIF considered important in the UAG discussion cannot simply be mapped onto the chapter structure of the NKLM. Even the learning objectives considered essential for PIF by the UAG can be found in several chapters. The desire to reformulate or reword the two central learning objectives also supports this interpretation.

On the other hand, the heterogeneity of the collection of learning objectives and the evaluations may also show that there is no consensus on how to define PIF. There are obviously different interpretations of what a PIF can be understood to mean and there may also be different answers to the question of whether an explicit curriculum for PIF with specially defined learning objectives is necessary, which would then have to be included in the catalog of learning objectives.

The committee's experience shows that both aspects should be taken into account in the further development of the PIF topic area. If PIF is to be explicitly represented in the NKLM, then the corresponding learning objectives would have to be formulated more precisely. The results presented in this article could serve as a starting point for this. In addition, a theory-based definition of PIF would also be helpful [15] in order to revise and, if necessary, supplement these PIF learning objectives, which are considered essential, and thus also to define the boundaries of the subject area more clearly. At the same time, there will probably still be some ambiguities, which are most likely inherent in the nature of the subject and must be resolved in practice in the actual design of curricula by finding compromises appropriate to the specific context [26].

The compilation of learning objectives presented here can also be used for this purpose: the learning objectives assessed as essential can form the core of a PIF curriculum, which must be explicitly communicated and presented in this sense. In contrast, the associated learning objectives offer the opportunity to address the vagueness of the learning objective field and also PIF as an overarching attitude. The teaching of PIF can thus reflect both aspects. Depending on the objectives of the individual curricula or courses, PIF can be focused on either as a subject area to be taught explicitly (essential learning objectives) or as a compilation of related topics and aspects (associated learning objectives). The compilation of learning objectives can be continuously supplemented on the basis of the work steps described in the UAG.

### 5.1. Limitations

Both the identification of the learning objectives relevant to PIF in the NKLM and the formulation of two fundamental learning objectives are limited by the fact that they are based on feedback from a small, self-selected group of people. In this respect, it cannot be ruled out that the participants have a one-sided or distorted view of the topic due to their previous experience and intensive engagement with PIF. On the other hand, previous experience and preconceptions could also contribute to greater awareness and thus enable a broader and more multi-layered discussion, which can be positive in exploratory work that we see more as the beginning of an ongoing process – especially since the NKLM is still a work in progress. 

Due to the limitation to the learning objectives of the NKLM, the number of topics and learning objectives was necessarily limited. However, it was precisely this examination of a limited number of learning objectives that enabled the UAG to sharpen its understanding of PIF.

The work described can only be beneficial if it is accompanied and expanded by practical experience with teaching PIF. The experience gained in this practice can then be incorporated into the development of guidelines and structures for dealing with PIF throughout the entire course of medical training.

## Acknowledgements

We would like to thank the members of the GMA Professional Identity Formation Committee for their constructive collaboration and helpful exchange on the further development of the list of learning objectives. 

## Authors’ ORCIDs

- Pascal O. Berberat: [0000-0001-5022-5265]
- Rolf Kienle: [0000-0003-2253-8832]
- Claudia Kiessling: [0000-0003-4104-4854]
- Daniela Mauer: [0009-0009-9469-793X]
- Goetz Fabry: [0000-0002-5393-606X]

## Competing interests

The authors declare that they have no competing interests. 

## Supplementary Material

Selected PIF learning objectives (as explained in the text)

NKLM learning objectives

## Figures and Tables

**Table 1:**
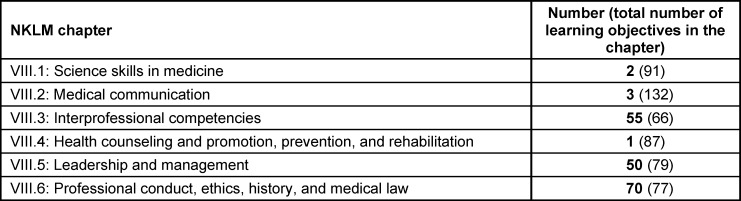
Number of learning objectives classified as relevant in the National Competency-Based Catalog of Learning Objectives for Medicine (NKLM, version 2.0) for the higher-order competencies for medical education (chapters VIII.1 to VIII.6). In parentheses: total number of learning objectives in the respective chapter

**Table 2:**
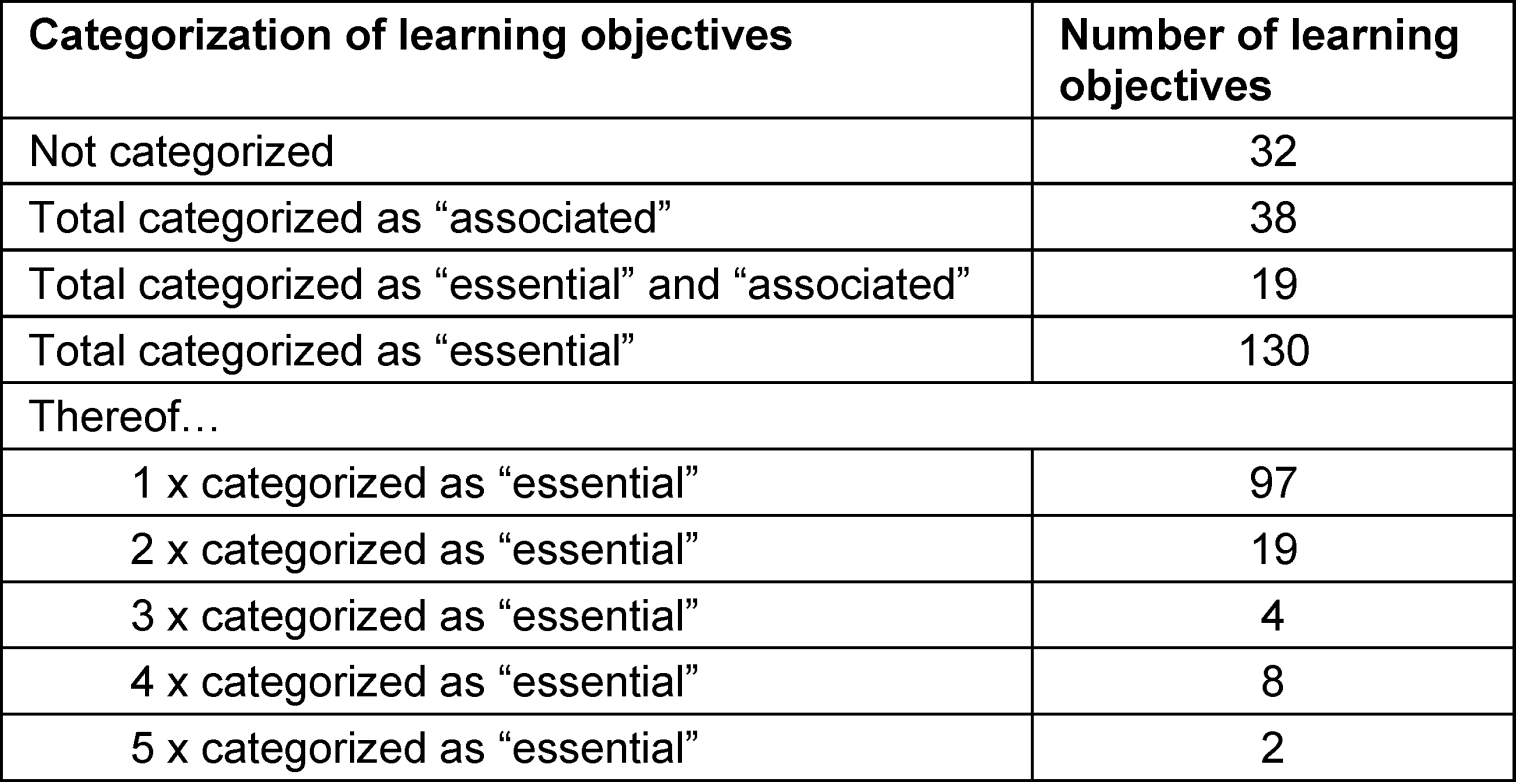
Number and categorization (“associated” vs. “essential”) of the selected learning objectives from the National Competency-Based Learning Catalog of Learning Objectives for Medicine (NKLM, version 2.0) relating to the higher-order competencies for medical education (chapters VIII.1 to VIII.6)
