# Supplementary material for: Professional identity formation (PIF) – What has NKLM 2.0 to say about it? A workshop report
Source: GMS J Med Educ. 2026 Mar 23;43(3):Doc34. doi: 10.3205/zma001828 (PMC13054817; doi:10.3205/zma001828)
Supplement: Selected PIF learning objectives (as explained in the text) [file JME-43-34-s-001.pdf]

Attachment 1: Selected PIF Learning Objectives (as explained in the text)

**Selected PIF Learning Objectives, NKLM (January 2023)**			**total selected**	**total „essential“**	**total „associated“**
**ID**	**No. LO**	**Competence / Sub-Competence / Learning Objective (LO)**			
VIII.1-01		**Central Concepts and Theories of Science: Graduates internalize the fundamentals of scientific thinking and action.**			
VIII.1-01.2		They master the principles of learning in the sense of recognizing and reflecting on their own learning needs, designing an adequate learning process based on these needs, and implementing learning outcomes. They are able to…			
VIII.1-01.2	12	Reflect on conflicts between their roles as a medical and scientific practitioner.	2		
VIII.1-02		**Graduates continuously review their professional knowledge and actions, identifying their own learning needs as part of a lifelong learning process.**			
VIII.1-02.1		They apply the fundamentals of the philosophy of science and research in the context of medicine. They are able to…			
VIII.1-02.1	6	Take for their continuous education to maintain and develop medical competencies by adequately assessing, evaluating, and, if necessary, pursuing appropriate measures for their development in various competency areas.	1	E 1	
VIII.2-02		**Graduates establish a trustworthy, stable doctor-patient relationship and master professional and patient-centered communication, considering specific conversation types, phases, and tasks.**			
VIII.2-02.1		They are capable of building and maintaining a positive, sustainable, and trustworthy doctor-patient relationship through their communication skills. They are able to…			
VIII.2-02.1	2	Adopt a patient-centered (congruent, accepting, and empathetic) attitude, communicate accordingly, and professionally manage closeness and distance.	1	E 1	
VIII.2-03		**Graduates reflect on typical sensitive topics in everyday medical practice and communicate appropriately even in emotionally challenging situations.**			
VIII.2-03.1		They are capable of recognizing emotions in patients and their caregivers and addressing communication disruptions accordingly. They are able to …			
VIII.2-03.1	2	Perceive emotions in interactions with patients, reflect on concepts of transference and countertransference, and maintain an appropriate and objective communication style.	2		
VIII.2-03.2		Identify and name typical sensitive topics that may arise in medical practice and conduct relevant discussions or consultations sensitively and in accordance with current standards.			
VIII.2-03.2	1	Manage general uncertainties of medical decisions and individual uncertainties, and communicate these appropriately to patients, considering their role and training level.	1	E 1	
VIII.3-01		**Graduates collaborate with various health professions based on mutual respect and shared values.**			
VIII.3-01.1		They align their actions in interprofessional health care with individual patient interests and their environment. They are able to…			
VIII.3-01.1.	1	Protect the dignity, autonomy, and privacy of treated individuals in interprofessional care.	1	E 1	
VIII.3-01.1.	2	Handle information confidentially in interprofessional care, considering essential data protection principles.	2	E 1	
VIII.3-01.1.	3	Apply ethical principles in the context of challenging situations, such as palliative and end-of-life care.	2	E 1	
VIII.3-01.1.	4	Recognize and name ethical dilemmas in the interprofessional care of children and non-consenting individuals, and develop solutions with the team, including with the person and other concerned parties.	2	E 1	
VIII.3-01.1.	5	Recognize and name ethical dilemmas in the interprofessional care of patients with mental disorders and develop solutions with the team, including with the person and other concerned parties.	1	E 1	
VIII.3-01.1.	6	Recognize and name ethical dilemmas in the interprofessional care of patients in gynecology and obstetrics, and develop with the team, including with the person and other concerned parties.	1	E 1	
VIII.3-01.1.	7	Recognize and name ethical dilemmas in conflict-ridden areas of care and develop solutions with the team, including with the person and other concerned parties.	1	E 1	
VIII.3-01.2		They consider the diversity of individual team members and patients, fostering value-oriented interactions. They are able to…			
VIII.3-01.2	1	Recognize sociocultural characteristics of team members in the interprofessional team and consider their potential impacts on the intended treatment goals.	3	E 2	
VIII.3-01.2	2	Recognize sociocultural characteristics of the individuals being treated in interprofessional collaboration and consider their potential impacts on the intended treatment goals.	1	E 1	
VIII.3-01.2	3	Adopt an open, appreciative, and respectful attitude within the interprofessional team and make this the foundation of the relationship, as well as initiate and maintain appropriate measures to establish this relationship.	3	E 2	
VIII.3-01.2	4	Maintain an, appreciative, and respectful attitude in stress-prone situations and during complex phases of patient care.	3	E 1	A 1
VIII.3-01.2	5	Adopt and sustain an open, appreciative, and respectful attitude during handovers.	3	E 1	A 1
VIII.3-01.2	6	Perceive emotions in interactions with colleagues, reflect on processes of transference and countertransference, and maintain an appropriate and objective communication style.	4	E 2	
VIII.3-02		**Graduates are able to explain their own role and the role of other health professions in promoting public health and caring for patients. They can apply this knowledge in health promotion and prevention, treatment, rehabilitation, and palliative care.**			
VIII.3-02.1		They explain the role of representatives of different professions in different institutions regarding health promotion, prevention, cure, rehabilitation, and palliative care. They are able to...			
VIII.3-02.1	1	reflect on and explain their own tasks, functions, and responsibilities with regard to cooperating with other	3	E 3	
VIII.3-02.1	2	Explain the tasks, role, and responsibilities of nurses.	1		A 1
VIII.3-02.1	3	Explain the tasks, role, and responsibilities of physiotherapists.	1		A 1
VIII.3-02.1	4	Explain the tasks, role, and responsibilities of occupational therapists.	1		A 1
VIII.3-02.1	5	Explain the tasks, role, and responsibilities of clinical social workers.	1		A 1
VIII.3-02.1	6	Explain the tasks, role, and responsibilities of psychological psychotherapists.	1		A 1
VIII.3-02.1	7	Explain the tasks, role, and responsibilities of pharmacists.	1		A 1
VIII.3-02.1	8	Explain the tasks, role, and responsibilities of medical receptionists.	1		A 1
VIII.3-02.1	9	Explain the tasks, role, and responsibilities of midwives.	1		A 1
VIII.3-02.1	10	Explain the tasks, role, and responsibilities of speech therapists.	1		A 1

VIII.3-02.1	11	Explain the tasks, role, and responsibilities of paramedics.	1		A 1
VIII.3-02.1	12	Explain the perception, delegation, and, where regulated, substitution of responsibility in collaboration with members of various medical and healthcare professions using clinical examples.	2		A 1
VIII.3-02.1	14	Identify, describe, and communicate their own limitations in terms of expertise, abilities, and skills in a manner appropriate to the situation.	4	E 2	
VIII.3-02.1	15	Consider the roles and responsibilities of other service providers and carriers in health promotion, prevention, cure, rehabilitation, and palliative care in interprofessional collaboration.	1		A 1
VIII.3-03		**Graduates communicate adequately as team members with representatives of different health professions to optimize collaboration and quality of care.**			
VIII.3-03.1		They are capable of building and maintaining a positive, sustainable, and trustworthy relationship within the team through their communication skills. They are able to…			
VIII.3-03.1	1	Communicate respectfully in an understandable and appropriate manner for all team members.	1	E 1	
VIII.3-03.1	2	Explain and utilize verbal and non-verbal communication methods and techniques, along with their advantages, disadvantages, and necessary conditions during teamwork.	1	E 1	
VIII.3-03.1	3	Explain and utilize digital communication methods and techniques, along with their advantages, disadvantages, and necessary conditions during teamwork.	1		
VIII.3-03.1	4	Provide timely, situational, and constructive feedback within the team.	1	E 1	
VIII.3-03.1	5	Accept feedback as a team member and respond appropriately.	1	E 1	
VIII.3-03.2		They develop and integrate error and safety culture within the team. They are able to…			
VIII.3-03.2.1	1	Explain team-based factors influencing safety culture.	1		
VIII.3-03.2.1	2	Explain the impacts of team dynamics and formation on interprofessionalism.	1	E 1	
VIII.3-03.2.1	3	Explain the significance of different perceptions of risks, cognitive biases, and risk propensity of team members.	1	E 1	
VIII.3-03.2.1	4	Identify adverse events and near misses within the team and communicate them in a situation-specific, timely, direct, and open manner.	1	E 1	
VIII.3-03.2.1	5	Explain human factors in the team in case of error occurrence.	1	E 1	
VIII.3-03.2.1	10	Explain preventable adverse events related to patient safety within the team.	1	E 1	
VIII.3-03.2.1	11	Analyze adverse events and near misses within the team.	1	E 1	
VIII.3-03.2.1	12	Collaboratively develop, implement, and evaluate solutions and measures to prevent errors.	1	E 1	
VIII.3-03.3		They develop and integrate a culture of error management and safety within the team. They are able to...			
VIII.3-03.3.1	1	agree on concrete solutions with all parties involved based on an analysis of team conflicts,	1	E 1	
VIII.3-03.3.1	2	Apply communication models to de-escalate potentially contentious conversations within the team.	1		
VIII.3-03.4		They communicate within the team to accomplish shared tasks. They are able to…			
VIII.3-03.4.1	1	Conduct patient handovers problem-focused, structured, and target-group-oriented to professionals in psychotherapy, nursing, physical therapy, clinical social work, nutritional counseling, and medical resceptionists, using appropriate technical language and media (including comprehension checks).	1	E 1	
VIII.3-03.4.1	2	Communicate patient-related information, including proper documentation, to health professions not directly involved in patient care, both within and outside their own, ensuring quality and safety.	1	E 1	
VIII.3-03.5		They communicate within the team to accomplish tasks together. They are able to...			
VIII.3-03.5.1	1	Name the necessity of comprehensive documentation for interprofessional teamwork and explain this based on clinical examples referencing medical information management.	1		
VIII.3-03.5.1	2	Document information about the treated individual and their situation target-group-oriented, accurately, and understandably.	1	E 1	
VIII.3-03.5.1	3	Incorporate and adequately use documentation from other professions for clinical-making.	1		
VIII.3-04		**Graduates can successfully act as team members to optimally shape health promotion, prevention, cure, rehabilitation, and palliation.**			
VIII.3-04.1		They reflect on and justify their intra- and interprofessional actions theoretically based on scientifically founded models. They are able to…			
VIII.3-04.1	1	Explain essential concepts of teamwork and group dynamics.	1	E 1	
VIII.3-04.1	2	Explain concepts/models of interprofessional collaboration and distinguish them from various forms of disciplinarity.	1		
VIII.3-04.2		They Involve representatives of different health professions various institutions in joint patient-centered needs assessment, planning, execution (problem-solving) of the care process intra- and intersectorally. They are able to…			
VIII.3-04.2	1	Gather and reflect on treatment-relevant information regarding the individual's biopsychosocial situation in the team.	1	E 1	
VIII.3-04.2	3	Design clinical decision-making processes in the team across professions and sectors, focusing on patient orientation.	1	E 1	
VIII.3-04.2	4	Develop individually relevant therapy and rehabilitation goals interprofessionally and jointly with patients and possibly their families.	1	E 1	
VIII.3-04.2	9	Adapt the formation of health professions involved in health promotion prevention, cure, rehabilitation, and palliation to developmental, age, and gender-specific differences.	1		
VIII.3-04.2	10	Reflect on aspects of interprofessional healthcare and care for children and adults with intellectual or multiple disabilities, considering common comorbidities, communication particularities, and the social environment of the affected	1		
VIII.3-04.2	11	Identify the health literacy of patients within the team and initiate joint measures to strengthen health literacy.	1		
VIII.4-04		**The graduates explain, reflect on, and advise on disease- and target group-specific measures of prevention, health promotion, and rehabilitation, taking individual aspects and participation into account**			
VIII.4-04.12		They identify and facilitate psychosocial assistance as preventive offerings. They are able to…			
VIII.4-04.12	3	Explain their role as mediators and cooperation partners within the interdisciplinary framework of psychosocial support services.	2		
VIII.5-01		**Graduates develop an understanding of their role as physicians.**			
VIII.5-01.1		They reflect on their own role as leaders and managers within the healthcare system. They are able to…			
VIII.5-01.1	1	Assess, reflect, and develop their future role as a physician in society and leaders.	5	E 5	
VIII.5-01.1.2	2	Recognize and develop their future role as a physician in the respective settings of prevention (primary, secondary, tertiary prevention), curation, rehabilitation, nursing, inpatient care, and basic, standard, or maximum care.	3	E 1	A 1

VIII.5-01.1	3	Derive and reflect on areas of frictions within the normative and social context for physicians.	4	E 4	
VIII.5-01.2		They reflect on and develop their individual medical personality and align their medical work with it. They are able to…			
VIII.5-01.2	1	Consciously adopt basic medical attitudes and align their medical actions.	4	E 4	
VIII.5-01.2	2	Recognize the competence of patients as experts themselves as equal to medical expertise for a successful healing process, perceive and name differences in competence, and align their medical actions accordingly.	2	E 2	
VIII.5-01.2	3	Reflect on, develop, and integrate a basic ethical stance that appreciates, considers, and promotes the ethical core values of patients across different confessions.	3	E 1	A 1
VIII.5-01.2	4	Reflect on the significance of their own medical personality for the development of a healing doctor-patient relationship.	3	E 3	
VIII.5-01.3		They develop a basic medical attitude of empathy and learn to be mindfully present for patients. They are able to…			
VIII.5-01.3	1	care for their patients in a humane manner without losing their objectivity, and distinguish between empathy and pity, while being aware of the supportive effect of emotional attention to patients.	4	E 4	
VIII.5-01.3	2	Communicate understandably with non-medical personnel, capable of medically and humanely engaging communication.	2	E 1	A 1
VIII.5-01.3	3	Reflect on the importance of situational and conscious non-action, medical observation (watchful waiting), and presence for the ill as an expression of therapeutic assistance.	2	E 2	A 1
**VIII.5-03**		**Graduates consider aspects of the economy in the healthcare system.**			
VIII.5-03.1		They explain essentially relevant concepts of health economics. They are able to…			
VIII.5-03.1	1	Name and describe economic principles (incentive systems) and their significance for their own medical actions.	2	E 2	
VIII.5-03.1	3	Describe the mutual influences of economic conditions and medical progress.	1		A 1
VIII.5-03.3		They analyze the health market They are able to…			
VIII.5-03.3	1	Name incentive structures, levels, and forms, and explain the effect of incentives.	2	E 1	A 1
VIII.5-04		**Graduates responsibly manage resource allocation.**			
VIII.5-04.1		They understand the essential concepts of resource allocation in the healthcare system. They are able to…			
VIII.5-04.1	1	Discuss implicit and explicit prioritization and its impacts.	1	E 1	
VIII.5-05		**Graduates are familiar with models and methods of quality management and apply them.**			
VIII.5-05.2		They know terms, concepts, and tools of quality management and implement them. They are able to…			
VIII.5-05.2	5	Actively engage in structured team meetings.	1	E 1	
VIII.5-05.2	6	Explain the principles of clinical risk management.	1	E 1	
VIII.5-06		**Graduates utilize time management.**			
VIII.5-06.1		They develop methods to structure and organize their working time. They are able to…			
VIII.5-06.1	1	Apply various methods for defining and prioritizing goals and tasks.	1		A 1
VIII.5-07		Graduates engage in career planning.			
VIII.5-07.1		They plan their own career and identify their personal qualification and support needs. They are able to…			
VIII.5-07.1	1	Identify potential support forms and funding opportunities for their career planning and explain their advantages and disadvantages.	2	E 1	A 1
VIII.5-07.1	2	Present the career options available to them after graduation, as well as the respective access to these fields, and evaluate the advantages and disadvantages of each option to apply them to their own career plan (theoretically).	1	E 1	
VIII.5-08		**Graduates develop leadership skills.**			
VIII.5-08.1		They know which tasks can be delegated and are able to delegate them. They are able to…			
VIII.5-08.1	1	Give clear, structured and understandable instructions that enable the commissioned person to work relatively independently.	1		
VIII.5-08.3		They know which tasks can be delegated and are able to delegate them. They are able to…			
VIII.5-08.3	1	Recognize and discuss various leadership styles and their impacts.	1		A 1
VIII.5-08.3	2	Assume leadership responsibility in a team in specific situations and adopt an appropriate leadership style.	2	E 1	A 1
**VIII.5-9**		**Graduates evaluate interfaces within the healthcare system.**			
VIII.5-9.2		They can manage interfaces in patient. They are able to…			
VIII.5-9.2	2	Discuss a specific patient in a situation-adapted consultative manner with colleagues.	1	E 1	
VIII.5-9.2	3	Define the characteristics of a complete patient handover and conduct it based on an example, considering interprofessional aspects.	1	E 1	
VIII.5-9.2	4	Recognize and apply the various types of medical documentation, their structures, and areas of application.	1	E 1	
VIII.5-10		**Graduates internalize a comprehensive concept of patient safety and align practical patient care with it.**			
VIII.5-10.1		In connection with patient safety, they address fundamental definitions and essential aspects concerning law and responsibility. They are able to…			
VIII.5-10.1	1	Define and differentiate critical incidents, harm, and errors (system failures), as well as terms like "mistake," "complication," "error chain," or "adverse event," and discuss them using a specific case.	1	E 1	
VIII.5-10.1	2	Explain the differences between responsibility, assuming responsibility, and potential culpability in the context of clinical actions with examples.	2	E 2	A 1
VIII.5-10.2		They know typical errors and risks that influence patient safety. They are able to…			
VIII.5-10.2	1	Name various types of errors, explain the factors and mechanisms of error and complication occurrence, and identify and illustrate them in a specific case example.	1	E 1	
VIII.5-10.2	2	Reflect on others behavior, recognize errors, and communicate them appropriately with colleagues, superiors, as well as patients and their relatives.	2	E 1	A 1
VIII.5-10.2	4	Explain the influence of hierarchical organizational structures on patient safety.	1		A 1
VIII.5-10.2	5	Describe the impact of unintended errors and critical care situations on their own health and the safety of future patients, considering preventive measures terms of self-care.	2	E 1	A 1
VIII.5-10.2	6	Critically reflect on the fact that errors can occur as early as the diagnosis and indication stage and discuss this using a specific example.	1	E 1	

VIII.5-10.3		They are familiar with measures to improve patient safety, in particular by reflecting on risks and errors. They are able to ...			
VIII.5-10.3	2	identify, classify, and appropriately address complications that have occurred, as well as name medical, organizational, and communicative consequences for damage control/prevention.	1	E 1	
VIII.5-10.3	3	explain and apply existing standards for increasing patient safety.	1		
VIII.5-11		**The healing physician personality: Graduates should explore the dimensions of the physician's personality, in particular the development of healing qualities, attitudes, and behaviors, and develop and internalize them.**			
VIII.5-11.1		They practice and develop substantial skills in self-reflection and self-awareness. They are able to…			
VIII.5-11.1	1	Engage with measures to enhance their own body awareness and improve their health-related behavior.	2	E 1	A 1
VIII.5-11.1	3	Differentiate between cognitive and emotional empathy, and become aware of the diagnostic and therapeutic effects of their resonance capability.	2	E 1	
VIII.5-11.1	4	Develop their own intentional and perceptual spaces to better and more broadly understand patients, using mindful and nonverbal-intentional engagement with them.	1	E 1	A 1
VIII.5-11.1	5	engage in self-reflection and perceive their own thoughts, thought patterns, and ruminations, and cultivate a better way of dealing with them for their own well-being and that of their patients	4	E 4	
VIII.5-11.1	6	reflect on themselves and develop their medical personality in terms of healing self-efficacy for the treatment of patients.	4	E 4	
VIII.5-11.1	7	Consider the interplay of physical, emotional, intellectual, and higher consciousness functions, and the inseparable connections of subject, object, and intersubjectivity for understanding patients, their illnesses, and related disease and healing factors.	2	E 2	
VIII.5-11.1	8	Adopt an open and multi-perspective receptive attitude that seeks to understand patients without prejudice or self-interest.	2	E 1	A 1
VIII.5-11.1	9	Grasp the complexity of patients' life and disease realities, as well as the health system.	2	E 2	
VIII.5-11.1	10	become aware of their own role as a physician, which influences their ability to interact and relate to patients, as well as accept their own personality traits and incorporate them into relationship constellations.	5	E 5	
VIII.5-11.1	11	Develop heightened awareness to engage with patients and their concerns with maximum mindfulness and presence.	1		
VIII.5-11.1	12	Analyze personal trigger and projection patterns, as well as traumas and shadow topics, that can impair a healing doctor-patient relationship. They integrate these themes to develop an open and healing attitude toward their patients.	2	E 1	
VIII.5-11.1	13	Become aware of personal of meaning, worldview, and religious or spiritual orientation, and learn to use them as self-care resources.	2	E 1	A 1
VIII.5-11.1	14	Recognize their own cognitive and experiential self-structure. They are capable of discerning their own psychological components in behavior and action and retracting them if necessary.	2	E 1	
VIII.5-11.1	15	Reflect on individual grounding possibilities, utilize them, and strengthen their internal resources.	1	E 1	
VIII.5-11.2		They recognize, reflect on, and consider the complexity and significance physical, psychological, social environment, meaning, worldview, and religious or spiritual orientation of ill people for a successful and profound healing process.<br>They understand healing as a processual event. They are able…			
VIII.5-11.2	1	Assess and recount individual and collective consciousness development, and apply this knowledge to doctor-patient encounters, collegial interactions, and the healthcare system in a broader view of situations.	2		A 2
VIII.5-11.3		They are familiar with all levels of the doctor-patient relationship and have internalized the principles of a healing encounter with ill people. They are able to…			
VIII.5-11.3	1	Consciously engage with patients on levels of meaning, significance, and meaning-making as support for a successful healing process.	2		A 5
VIII.6-01		**Graduates align their actions with the fundamental values and norms of the profession.**			
VIII.6-01.1		They know and consider profession-specific ethical and legal foundations and have a historically informed understanding of the cultural-societal embedding of the medical profession and its practice. They are able to…			
VIII.6-01.1	1	Name concepts of human images and apply them to the practical context.	3	E 2	
VIII.6-01.1	2	explain the origins of medical ethics codes, in particular the Hippocratic Oath and the Geneva Declaration, and critically assess their significance for the current understanding of the medical profession and in a legal context.	5	E 3	
VIII.6-01.1	3	Reflect on the tension between the individual and society in different political systems.	3	E 1	
VIII.6-01.1	4	Explain the development and establishment of central values as well as ethical and legal norms in their socio-cultural and historical specificity and changeability.	4	E 2	
VIII.6-01.1	5	Describe the behavior of physicians in response state expulsion and disenfranchisement of colleagues under National Socialism.	1		
VIII.6-01.1	6	Explain the medical crimes under National Socialism and their aftermath post-1945.	1		
VIII.6-01.1	7	Explain important ethical basic terms, the tasks of ethics, and the relationship of morality, ethics, and law in their significance for medicine.	2	E 1	
VIII.6-01.2		They orient their actions towards central values and profession-relevant norms. They are able to...			
VIII.6-01.2	1	Identify the general medical duties and professional obligations, medical independence, treatment principles, as well as incompatibilities of medical practice, and orient their actions accordingly.	1	E 1	
VIII.6-01.2	2	Identify the legal stipulations regarding documentation obligation, access to patient records, and data protection, and orient their actions accordingly.	1	E 1	
VIII.6-01.2	3	Transparently communicate the prospects of success, alternatives, and risks of medical measures.	1	E 1	
VIII.6-01.2	4	Identify the legal regulations for proper examination and treatment methods, well as the criminal and liability consequences, and orient their actions accordingly.	1		
VIII.6-01.2	5	Explain and reflect on the various interests under which their actions take place in clinical practice and medical research, identify related potential conflicts, reflect on them, and consider them in their decisions.	3	E 1	
VIII.6-01.2	6	Identify the prerequisites and civil law regulations for the treatment contract and orient their actions accordingly.	1		
VIII.6-01.2	7	Treat patients respectfully, maintaining their autonomy, and align their actions with patients' individual values and needs.	1	E 1	
VIII.6-01.2	8	Orient their actions with respect to confidentiality and privacy.	1	E 1	
VIII.6-01.2	9	Recognize, analyze ethical conflicts, and professionally manage them in practice.	1	E 1	
VIII.6-01.2	10	Identify the medical criminal law norms and collateral medical criminal law norms and orient their actions accordingly.	1		
VIII.6-01.2	11	Identify the ethical, historical, and fundamental rights references, especially to human rights, the dignity of human beings, physical integrity, and the right to informational self-determination, and align their actions accordingly.	1	E 1	
VIII.6-01.3		They are capable of handling role-related challenges. They are able to...			
VIII.6-01.3	1	Define the medical responsibility area and orient their actions accordingly.	4	E 2	
VIII.6-01.3	2	Establish appropriate relationships in their professional role with patients, relatives, colleagues, nurses, and other professional groups.	4	E 2	
VIII.6-01.3	3	Distinguish between private and professional contexts or roles.	4	E 2	

VIII.6-01.3	4	Collaborate interdisciplinarily and interprofessionally with others, aiming for optimal patient treatment while neglecting professional political aspects.	4	E 1	
VIII.6-01.3	5	Name the legal requirements for delegating medical measures to non-medical health professions and medical students, considering assumption of and division of labor, and orient their actions accordingly.	1		
VIII.6-02		**The graduates consider socially relevant framework conditions in their actions.**			
VIII.6-02.1		They know and consider the characteristics, tasks, responsibilities, and development of the medical profession. They are able to…			
VIII.6-02.1	1	Explain the characteristics of a liberal profession, the structures and functions of the professional organization, as well as the Professional Code for Physicians..	2	E 2	
VIII.6-02.1	2	Explain the development and political role of the medical profession and evaluate the function and significance of professional politics in complex socio-political structures with historical context.	2	E 1	
VIII.6-02.1	3	Identify and explain the duties and roles of the responsible medical associations in professional practice, continuing education, and professional jurisdiction.	1		
VIII.6-02.2		They understand the framework conditions of healthcare. They are able to…			
VIII.6-02.2	1	Explain the historical developments of the healthcare system and their ethically relevant differences.	1		
VIII.6-02.2	2	Reflect on important ethical aspects of population-based measures for health, prevention, and disease control (public health).	1	E 1	
VIII.6-02.2	3	Reflect on the socioeconomic factors influencing health and the resulting inequalities in the health status of population groups.	1	E 1	
VIII.6-02.3		They understand and consider aspects of justice in healthcare. They are able to…			
VIII.6-02.3	1	Explain different concepts of justice and their consequences for medicine.	1	E 1	
VIII.6-02.3	2	Explain and on different strategies for dealing with limited resources in healthcare (and their medical, ethical, legal, and economic implications).	1	E 1	
VIII.6-02.3	3	Differentiate between different levels of allocation and assess the foundations of allocation decisions at different levels.	1		
VIII.6-03		**Graduates understand and consider personal needs and prerequisites in professional action.**			
VIII.6-03.1		They are capable of self-awareness, self-reflection, self-criticism, and self-development. They are able to…			
VIII.6-03.1	1	Observe and critically reflect on themselves and their actions.	5	E 4	
VIII.6-03.1	2	Realistically assess their own competencies and align the assumption of tasks and responsibilities accordingly.	5	E 1	
VIII.6-03.1	3	Recognize typical sources of errors related to individuals, teams, and communication, and apply specific strategies to avoid them.	3	E 2	
VIII.6-03.1	4	Provide and accept objective criticism, reflect on it, and potentially change their behavior.	4	E 4	
VIII.6-03.1	5	Analyze their own strengths and weaknesses and reflect on their personality and competencies accordingly.	5	E 4	
VIII.6-03.1	6	identify strategies and constructively manage their own uncertainty, fears, weaknesses, mistakes.	5	E 3	
VIII.6-03.1	7	Reflect on their moral position, develop it further, and defend it argumentatively.	5	E 2	
VIII.6-03.2		They consider health and well-being as prerequisites for professional practice. They are able to…			
VIII.6-03.2	1	Recognize potential signs of physical and psychological stress to consider the limits of their own resilience and the burdens of others in medical practice.	3	E 1	A 1
VIII.6-03.2	2	Employ individual strategies for coping with and reducing stresses and explain appropriate professional support services.	2	E 2	
VIII.6-03.2	3	Reflect on and consider that doctors are perceived as role models concerning health-related behaviors.	3	E 1	
VIII.6-04		**Graduates are familiar with ethical and legal questions in patient care.**			
VIII.6-04.1		They consider cultural, social, and life-world aspects in their actions. They are able to…			
VIII.6-04.1	1	Explain the special challenges and needs of people with rare diseases, particularly regarding diagnosis, therapy, medical care, research, and self-help.	1		
VIII.6-04.1	2	Consider diversity-related aspects of patients.	1	E 1	
VIII.6-04.1	3	Consider the individual's perceptions of health and illness of the patient in their actions and be able to adopt perspectives.	1	E 1	
VIII.6-04.1	3	Align their medical practice with particularly vulnerable groups.	1	E 1	
VIII.6-04.2		Respect patient autonomy appropriately. They are able to…			
VIII.6-04.2	1	Explain how socio-cultural, historical, legal, and political factors influence the understanding of patient autonomy.	2	E 1	
VIII.6-04.2	3	Explain the ethical and legal foundations of the principle of patient autonomy.	2	E 1	
VIII.6-04.2	4	Align their actions with ethical requirements and the legal regulations for medical clarification and consent by the patient, and name the criminal and liability consequences.	1		
VIII.6-04.2	5	Assess the special ethical and legal challenges of patient requests are not primarily oriented toward health purposes.	1	E 1	
VIII.6-04.2	7	Assess the particular challenges of substitute decision-making for patients who are not (or no longer) capable of giving consent and apply the ethical foundations and legal regulations in practice.	1	E 1	
VIII.6-04.2	8	Apply the ethical and legal conditions for medical coercive measures.	1	E 1	
VIII.6-04.3		They understand consider the importance and responsible handling of trust in doctor-patient relationships. They are able to…			
VIII.6-04.3	1	Apply measures for building trust responsibly.	3	E 1	A 1
VIII.6-04.3	2	Name different ethical models of the doctor-patient relationship and their historical and socio-cultural variability.	2		A 1
VIII.6-04.3	3	Explain and consider in their actions the specific requirements, challenges, opportunities, and boundaries of the doctor-patient relationship through new technological procedures.	2	E 1	A 1
VIII.6-04.4		They understand and consider ethical, social, cultural, legal, and historically relevant aspects in special medical care contexts. They are able to…			
VIII.6-04.4	1	Consider the essential legal frameworks and ethical challenges, as well as the historical and socio-cultural variability of attitudes toward and handling of reproduction and prenatal life.	1	E 1	
VIII.6-04.4	3	Consider ethical, social, cultural, legal, and historical aspects in the interaction with people with disabilities and their caregivers.	1	E 1	
VIII.6-04.4	4	Identify and consider essential ethical, legal, social, cultural, and historical aspects related to the treatment of children and adolescents.	1	E 1	
VIII.6-04.4	5	Identify relevant (fundamental) rights references to child protection and align their actions, e.g., when breaking medical confidentiality for the child's welfare, and initiate appropriate measures in cases of suspected child endangerment.	1	E 1	
VIII.6-04.4	6	Name present ethical, social, cultural, and historical aspects, as well as the legal framework of human genetic diagnostics and therapy.	1		

VIII.6-04.4	7	Reflect on essential ethical, legal, social, historical, and cultural aspects in the area of illness.	1	E 1	
VIII.6-04.4	8	Present the essential ethical, social, legal, cultural, and historical in the field of transplant medicine, particularly organ donation.	1	E 1	
VIII.6-04.4	9	Present the ethical, legal, social, and culturally relevant aspects in the last phase of life and consider them in the interaction with patients and their caregivers.	1	E 1	
VIII.6-04.4	10	Assess the historical development of the euthanasia debate in the context of current discussions.	1	E 1	
VIII.6-04.4	11	Explain the genesis and development of historical and current, including legal, definitions of death and assess their socio-cultural conditions and ethical implications.	1		
VIII.6-04.4	12	Explain the, tasks, and working methods of ethics consultations and ethics committeesRecognize and address disadvantages, stigmatization, and discrimination based on race, ethnicity, gender, religion or belief, disability, age, or sexual identity to prevent or eliminate these disadvantages.	1		
VIII.6-04.4	13	Name and consider the essential ethical challenges of big data-supported medicine and the legal framework.	1	E 1	
VIII.6-04.4	14	Name ethical foundations and drug law regulations, particularly on drug requirements and application, as well as approval or off-label use and drug repurposing for human protection in clinical testing and liability for drug damage, and align their actions accordingly.	1		
VIII.6-04.4	16	Reflect on the challenges of digitalization in the context of clinical practice and research.	1		
VIII.6-04.4	17	Consider the essential legal frameworks and ethical challenges, as well as the historical and socio-cultural variability of attitudes toward and handling of reproduction and prenatal life.	1		
