# Supplementary material for: Professional identity formation (PIF) – What has NKLM 2.0 to say about it? A workshop report
Source: GMS J Med Educ. 2026 Mar 23;43(3):Doc34. doi: 10.3205/zma001828 (PMC13054817; doi:10.3205/zma001828)
Supplement: NKLM learning objectives [file JME-43-34-s-002.pdf]

Attachment 2: NKLM learning objectives

The learning objectives were marked as “essential for the PIF” by at least 3 out of 6 experts. For a better understanding, the competence or sub-competence (highlighted in gray) to which the learning objectives belong is also listed in italics. The number of experts who selected the respective learning objective as PIF-relevant (N=6) from all learning objectives in chapters VIII.2 – VIII.6 in the previous step (see 2.2) is also indicated.

NKLM 2.0 ID	Competence / Sub-Competence / Learning Objective	Classified “PIF-relevant”	Classified “essential for PIF”
VIII.3-02	*Graduates are able to explain their own role and the role of other health professions in promoting public health and caring for patients. They can apply this knowledge in health promotion and prevention, treatment, rehabilitation, and palliative care.*		
VIII.3-02.1	*They explain the role of representatives of different professions in different institutions regarding health promotion, prevention, cure, rehabilitation, and palliative care. They are able to...*		
VIII.3-02.1.1	reflect on and explain their own tasks, functions, and responsibilities with regard to cooperating with other health professions.	**3**	**3**
VIII.5-01	*Graduates develop an understanding of their role as physicians.*		
VIII.5-01.1	*They reflect on their own role as leaders and managers in the healthcare system. They are able to...*		
VIII.5-01.1.1	assess, reflect on, and develop their own future role as doctors in society and as leaders.	**5**	**5**
VIII.5-01.1.3	Identify and reflect on areas of friction regarding the normative and social context for physicians.	**4**	**4**
VIII.5-01.2	*They reflect on and develop their individual medical personality and align their medical work with it. They are able to...*		
VIII.5-01.2.1	consciously adopt basic medical attitudes and align their medical actions with them.	**4**	**4**
VIII.5-01.2.4	Reflect on the significance of their own medical personality for the development of a healing doctor-patient relationship.	**3**	**3**
VIII.5-01.3	*They develop a basic medical attitude of empathy and learn to be mindfully present for patients. They are able to…*		
VIII.5-01.3.1	care for their patients in a humane manner without losing their objectivity, and distinguish between empathy and pity, while being aware of the supportive effect of emotional attention to patients.	**4**	**4**
VIII.5.11	*The healing physician personality: Graduates should explore the dimensions of the physician’s personality, in particular the development of healing qualities, attitudes, and behaviors, and develop and internalize them.*		
VIII.5-11.1	*They practice and develop substantial skills in self-reflection and self-awareness. They are able to...*		
VIII.5-11.1.5	engage in self-reflection and perceive their own thoughts, thought patterns, and ruminations, and cultivate a better way of dealing with them for their own well-being and that of their patients.	**4**	**4**
VIII.5-11.1.6	reflect on themselves and develop their medical personality in terms of healing self-efficacy for the treatment of patients.	**4**	**4**
VIII.5-11.1.10	become aware of their own role as a physician, which influences their ability to interact and relate to patients, as well as accept their own personality traits and incorporate them into relationship constellations.	**5**	**5**

Attachment 2 to Ahles J, Apondo S, Berberat PO, Jörmann B, Kienle R, Kiessling C, Mauer D, Fabry G. Professional Identity Formation (PIF) – What has NKLM 2.0 to say about it? A workshop report. GMS J Med Educ. 2025;43(3):Doc34. DOI: 10.3205/zma001828

NKLM 2.0 ID	Competence / Sub-Competence / Learning Objective	Classified “PIF-relevant”	Classified “essential for PIF”
VIII.6-01	*Graduates align their actions with the fundamental values and standards of the profession.*		
VIII.6-01.1	*They know and consider profession-specific ethical and legal foundations and have a historically informed understanding of the cultural-societal embedding of the medical profession and its practice. They are able to…*		
VIII.6-01.1.2	explain the origins of medical ethics codes, in particular the Hippocratic Oath and the Geneva Declaration, and critically assess their significance for the current understanding of the medical profession and in a legal context.	**5**	**3**
VIII.6-03	*Graduates understand and consider personal needs and prerequisites in professional action.*		
VIII.6-03.1	*They are capable of self-awareness, self-reflection, self-criticism, and self-development. They can...*		
VIII.6-03.1.1	observe and critically reflect on themselves and their actions.	**5**	**4**
VIII.6-03.1.4	Provide and accept objective criticism, reflect on it, and potentially change their behavior.	**4**	**4**
VIII.6-03.1.5	Analyze their own strengths and weaknesses and reflect on their own personality and competencies accordingly.	**5**	**4**
VIII.6-03.1.6	identify strategies and constructively manage their own uncertainty, fears, weaknesses, mistakes.	**5**	**3**
